# Neural Pathways Conveying Novisual Information to the Visual Cortex

**DOI:** 10.1155/2013/864920

**Published:** 2013-06-06

**Authors:** Wen Qin, Chunshui Yu

**Affiliations:** ^1^Department of Radiology and Tianjin Key Laboratory of Functional Imaging, Tianjin Medical University General Hospital, Tianjin 300052, China; ^2^School of Medical Imaging, Tianjin Medical University General Hospital, Tianjin 300052, China

## Abstract

The visual cortex has been traditionally considered as a stimulus-driven, unimodal system with a hierarchical organization. However, recent animal and human studies have shown that the visual cortex responds to non-visual stimuli, especially in individuals with visual deprivation congenitally, indicating the supramodal nature of the functional representation in the visual cortex. To understand the neural substrates of the cross-modal processing of the non-visual signals in the visual cortex, we firstly showed the supramodal nature of the visual cortex. We then reviewed how the nonvisual signals reach the visual cortex. Moreover, we discussed if these non-visual pathways are reshaped by early visual deprivation. Finally, the open question about the nature (stimulus-driven or top-down) of non-visual signals is also discussed.

## 1. Introduction

The visual cortex has been traditionally considered as a stimulus-driven, unimodal system with a hierarchical organization, in which the early visual areas (V1, V2) tune to general features while the higher-tier ones (V3A, V4v, V7, hMT+, and V8) respond selectively to the specific features of a visual stimulus [1–5]. Two parallel visual streams have been proposed to generalize the hierarchical organization of the visual processing [6–8]. The dorsal stream or “where” pathway serves to analyze visual spatial information about object location, motion, and visuomotor planning. In this pathway, visual signals are conveyed to the posterior parietal cortex through the dorsal part of the visual cortex (such as the V3d, V3A, V7, and hMT+) and finally reach the prefrontal cortex. The ventral stream or “what” pathway has been associated with the processing of form, object identity, and color. This pathway conveys visual signals along the ventral part of visual cortex (such as VP, V4, and V8), the inferior temporal (IT) areas, and finally to the prefrontal cortex. 

The structural and functional organization of the visual areas is supposed to develop through a combination of genetic instruction [9–11] and experience-dependent refinement [12, 13]. The role of visual experience in the development of the visual areas is supported by a large number of neuroimaging studies revealing that the visual areas of congenitally blind (CB) and early blind (EB) subjects have increased cortical thickness [14–17], local brain spontaneous activity [18], metabolism, and blood flow [19–22] and decreased regional volume [23–25], white matter integrity [26, 27], anatomical network efficiency [28, 29], and altered resting-state functional connectivity (rsFC) [30, 31]. Moreover, converging evidence suggests that both the early and higher-tier visual areas in CB subjects are recruited during performing a variety of tasks given through nonvisual sensory modalities, as detailed in previous reviews [32–36]. 

However, the notion of the visual cortex as a unimodal system molded only by visual experience has recently been challenged because the visual cortex of both the sighted controls (SC) and the CB responded to a variety of nonvisual perceptive stimuli, including tactile, auditory, and olfactory. Furthermore, the visual cortex of the CB was also involved in cognitive processes, such as linguistic processing, working memory, and attention. Although the extent and magnitude of the activation in the visual areas depend on the tasks and subjects' characteristics [37, 38], the coactivation of several visual areas by nonvisual tasks in SC and CB highly indicates that the development of the functional organization of these visual areas does not require visual experience. 

The main topic of this review is to elucidate how the nonvisual signals recruit the visual cortex. We firstly provide evidence if the visual cortex is supramodal in nature, and then we reviewed how the nonvisual signals reach the visual cortex. Next, we discussed if these nonvisual pathways are reshaped by early visual deprivation. Finally, we also discussed about the nature (stimulus-driven or top-down) of nonvisual signals.

## 2. The Supramodal Nature of Visual Cortex

### 2.1. Cross-Modal Processing of Nonvisual Signals in the Visual Cortex in Sighted Subjects

#### 2.1.1. Tactile Stimuli Activate the Visual Cortex in Sighted Subjects

Unimodal theory supposes that the visual cortex is specifically allocated to process visual stimuli in sighted people; however, this hypothesis has recently been challenged. Using the positron emission tomography (PET), Sathian et al. first reported that the extrastriate area close to the parietooccipital fissure (V6) was activated during discrimination of grating orientation compared with discrimination of grating groove width, suggesting this visual area is recruited in the processing spatial information of tactile signals [39]. To further confirm that the occipital area is functionally involved in nonvisual processing, the transcranial magnetic stimulation (TMS) technique was used to transiently disrupt the functioning of this area. The authors found impaired tactile discrimination of grating orientation after exerting TMS on this occipital area and concluded that this occipital area is really functionally involved in tactile spatial perception [40]. Since then, many studies have reported the involvement of visual areas in a series of tactile processing, including the hMT+ complex for tactile motion perception [37, 41] and the ventral visual pathway for tactile object discrimination [42–49]. It is interesting to note that the hMT+ is capable of processing motion-related information even when the stimulus is delivered to the tongue [50]. Furthermore, several studies using both visual and tactile stimuli showed that these two modalities drive the same visual areas for motion and object processing, supporting the cross-modal involvement of visual areas in the abstract representation of the concepts of objects, space, and motion [38, 42].

#### 2.1.2. Auditory Stimuli Activate the Visual Cortex in Sighted Subjects

The cross-modal recruitment of the visual cortex has also been reported in auditory domain. As early as 1972, Morrell found that up to 41% of recorded neurons in extrastriate cortex of adult cats were modulated by both visual and auditory stimuli and that the receptive fields for both responses typically overlapped in space [51]. Recent studies in humans have also provided evidence of occipital activation in auditory processing [52, 53]. For example, the hMT+ complex was activated in sighted subjects while listening to auditory motion stimuli [52, 54, 55]. In accordance with the neuroimaging findings, several TMS studies showed that transient disruption of the specific occipital regions can impair the auditory perception in sighted subjects, such as inhibition of the extrastriate cortex induced a systematic error in auditory spatial perception [56], and disruption of the dorsal extrastriate cortex impaired the sound localization [57, 58]. The TMS evidence supports that the visual cortex is involved in spatial hearing in sighted subjects. It is also interesting to note that the visual cortex in sighted subjects cannot only percept the sound itself but also response to abstract auditory information such as action sounds [59]. 

### 2.2. Cross-Modal Processing of Nonvisual Signals in the Visual Cortex in Early Blind Subjects

Relative to the sighted subjects, cross-modal processing of nonvisual signals in the visual cortex has been more extensively reported in the CB and EB subjects when they perform nonvisual perception and high-order cognitive tasks. 

#### 2.2.1. Tactile Perception

Numerous studies reported that the visual cortex was recruited during diverse tactile tasks, such as the early and higher visual areas were activated in vibrotactile frequency discrimination [60], the hMT+ in tactile motion perception [37, 50, 61], and the ventral visual pathway in tactile object perception [38]. The visual cortex is also involved in tactile perception of the tongue [42, 62, 63]. Using the tongue display device (TDU), a tactile-to-vision sensory substitution device that translates a visual image into electrotactile stimulation, several studies have shown that the specific visual areas were recruited to process different types of tongue tactile stimuli, such as the ventral stream for tactile-form recognition [42], the hMT+ complex for tactile motion discrimination [50], and the ventral lateral occipitotemporal cortex for virtual route recognition [62]. Moreover, rTMS inhibition of the human hMT+ impaired the tactile speed discrimination, indicating that the recruitment of hMT+ is necessary for tactile motion processing [41]. Interestingly, TMS stimulation of the visual cortex can induce subjective tongue-tactile sensations in the CB who is proficient at the use of the TDU, which indicates that the perceptual correlate of activity in the visual cortex reflects the characteristics of its novel sensory input source [64].

#### 2.2.2. Auditory Perception

Similar with the tactile perception, the activation of the visual cortex by auditory perception was also frequently reported in CB subjects [54, 65, 66]. In an early PET study, Weeks et al. [67] reported that the right dorsal visual cortex was activated by auditory perception task in the CB but not in the SC. This region was also activated in EB subjects by an auditory spatial processing task using a sensory substitution prosthesis translating visual information into sounds [65]. The activation pattern in EB was also confirmed by recent studies [66, 68, 69]. Accordingly, the visual areas previously considered to be involved in visual motion processing (such as the hMT+) were specifically recruited in the EB by motion stimuli presented through the auditory modality [50, 54, 55, 70, 71]. The ventral pathway can be recruited to process auditory object recognition [72, 73] in the CB. In a recent study, Striem-Amit et al. [74] showed that the dorsal stream processed the location information, whereas the ventral stream responded to shape information via the vision-to-sound substitutes in CB subjects. The dorsal and ventral pathway recruited by auditory perception was further confirmed by electrophysiological and TMS evidence. Using ERP, several groups showed greater amplitude of the N1 component at the visual region in sound localization, suggesting the involvement of the visual cortex in early auditory processing in the EB [75–77]. TMS inhibition of the visual area can impair specific auditory performance in the EB [57, 78, 79]. For example, rTMS delivered to right dorsal extrastriate cortex disrupted the spatial processing of sounds in the EB [57, 78], and rTMS over the LOC can impair a EB subject's ability to identify objects [79]. 

#### 2.2.3. Olfactory Sensation

In the CB, besides auditory and tactile perception, the visual cortex was also recruited in olfactory processing [80]. In this study, a simple odor detection task, the authors found that CB subjects not only showed strong activation in the olfactory cortex, but also showed widespread activation in the visual cortex [80]. Combining earlier studies reported that superior olfactory perception in the CB [81, 82]; the recruitment of the visual cortex during odor detection suggests a preferential access of olfactory stimuli to this area in the CB.

#### 2.2.4. Cognitive Processing

The visual cortex in EB subjects is not only involved in nonvisual perception, but also takes part in the process of higher-level cognitive tasks, such as language, attention, and working memory. Converging evidence supports the involvement of the visual cortex in language processing in the CB or EB subjects. The medial visual cortex was recruited during Braille reading [83–85] and the occipitotemporal visual areas were activated during covert verb generation in the EB [86, 87]. Further studies reveal that the visual cortex is preferentially recruited by semantic relative to phonological processing [88], and the magnitude of fMRI activation is associated with both semantic and syntactic complexity [89]. A recent study showed that the visual word form area (VWFA), a component of the ventral stream that develops expertise for visual reading, can also process Braille reading in the EB [90]. Further evidence for the involvement of the visual cortex in language processing in the EB has been provided by a combination of task activation and functional connectivity analyses [91]. The authors found that (1) the responses of the visual regions and classic language regions across conditions were similar; (2) language sensitivity was restricted to the left visual cortex; (3) the left visual regions that responded to language had increased functional connectivity with classic language regions [91]. Besides the neuroimaging findings, in the CB, disruption of the visual cortex by TMS or lesions impairs the performance of Braille reading and verb generation [84, 92–95].

 Besides the language processing, the visual cortex that normally subserves vision is activated in the CB subjects when performing nonvisual attention-demanding tasks, such as spatial attention discrimination [32, 35, 36]. More importantly, the amplitude of the occipital activation in the CB was correlated with the spatial attention performance [66, 96, 97]. These findings suggest that the occipital activation is associated with the enhanced nonvisual attention abilities in the CB. 

The visual cortex can also be activated by memory task with nonvisual stimuli or without any sensory input [98–101]. The posterior occipital region (including V1) was recruited during a verbal memory task even without real sensory stimulation in the CB, and the activation magnitude of this region was correlated with verbal memory performance [101]. Bonino et al. reported that tactile spatial working memory task activated the dorsal extrastriate areas in the CB individuals [100]. Moreover, using three different kinds of working memory tasks (verbal, tactile, and auditory), a recent study showed that the visual cortex of the EB subjects responded to all types of stimuli [99]. 

### 2.3. Supramodal versus Plastic Mechanisms of the Occipital Activation

Two neural mechanisms have been proposed to explain the involvement of the visual cortex in the processing of nonvisual stimuli. One hypothesis holds that the visual cortex is supramodal in nature, which means that an occipital area relies on a common, abstract representation of the perceived stimuli irrespective of the sensory modality. Another hypothesis is the cross-modal plasticity. In the CB or EB, the visual cortex that normally serves to process visual input shifts to cross-modal process nonvisual information via plastic reorganization of the inner structure and function. However, the two mechanisms are not mutually exclusive, and they might coexist in the EB. 

As discussed above, many pieces of evidence that showed the involvement of the visual areas in processing nonvisual inputs in both the SC and EB may support the supramodal hypothesis (see Sections 2.1 and 2.2). The cross-modal involvement of the visual cortex in processing nonvisual stimuli means that the functional specialization of the visual areas is task-dependent rather than sensory modality-dependent. Furthermore, this pattern cannot be fully explained by visual imagery [102–108] because the CB subjects who never have visual experience also show occipital response to the nonvisual stimuli [69, 80]. The supramodal hypothesis can explain that visual experience is not necessary to develop the normal functional organization of the visual areas, which has been confirmed in a variety of previous studies on the CB [74, 90, 109, 110], because the development of the functional organization may be driven by inputs from other sensory modalities. 

The cross-modal plasticity is supported by the following evidence. The CB subjects commonly showed superior performance during auditory or tactile perception than normal sighted subjects [97, 101, 111–115]. The superior performance has also been associated with the occipital activation in the CB [96, 97, 101], suggesting the hypothesis of the cross-modal plasticity. This mechanism can also be applied to explain the involvement of the visual cortex in the higher-level cognitive tasks in the CB but not in the SC [84, 92–95]. Furthermore, the plasticity mechanism may also partly explain the increased cortical thickness [14–17], local brain spontaneous activity [18], metabolism and blood flow [19–22], and rsFC [30, 31] in the EB. 

Studies on the functional characteristics of the hMT+ have well described the coexistence of the two mechanisms in the EB. In sighted subjects, the hMT+ complex is segregated into an anterior part (supramodal region), that is, involved in processing both visual and tactile motion, and a posterior part (unimodal region), which is only involved in processing visual information. In the EB, however, the entire hMT+ is involved in the representation of tactile motion, suggesting the coexistence of the supramodal (anterior part) and plastic (posterior part) mechanisms in this region. These results represent competitive interactions between visual and nonvisual inputs in the reshape of hMT+ complex [37, 116]. 

It should be noted that the improved nonvisual perception performance in the early blind subjects can also be the consequences of the experience-dependent plasticity of their auditory or somatosensory related cortices. For example, the mice that are binocularly enucleated from birth demonstrate remarkable expansion of their barrel cortex, which may be interpreted by increased usage of the whiskers after visual deprivation [117]. Cats that were deprived of vision from birth also show expanded primary somatosensory and auditory areas; furthermore, the neurons in the anterior ectosylvian visual area (AEV) that normally respond to visual stimuli are replaced by neurons of neighboring auditory ectosylvian area (AEA), which is accompanied by the improvement of auditory spatial tuning of this region than sighted controls [118, 119]. As a result, the improved auditory/tactile perceptive performance might both be caused by the experience-dependent plasticity of the classic auditory/tactile regions with expanded cortical area, and by classic visual regions that turn to subserve the nonvisual information. Caution should be paid that some cortical regions such as the “AEV” in the congenitally blind subjects are actually replaced by expanded auditory cortices and their activation by auditory stimuli cannot be interpreted as cross-modal involvement of visual areas any more.

## 3. The Candidate Pathways That Nonvisual Signals Reach the Visual Cortex

**Figure 1:**
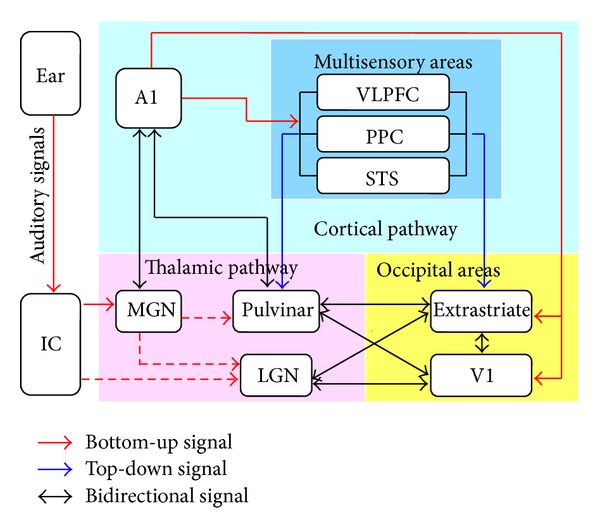
Schematic of neural pathways that convey auditory signals into visual areas. The solid line represents the existing connections in the normal sighted animals or human; dash line represents the rewired connections after early visual deprivation; arrows with red, blue, and black color represent the bottom-up, top-down, and bidirectional auditory signals, respectively. All these connections are confirmed by previous animal or human studies (for details see Section 3). A1: primary auditory cortex; IC: inferior colliculus; MGN: medial geniculate nucleus; LGN: lateral geniculate nucleus; PPC: post parietal cortex; STS: superior temporal cortex; VLPFC: ventral lateral prefrontal cortex; V1: primary visual cortex.

**Figure 2:**
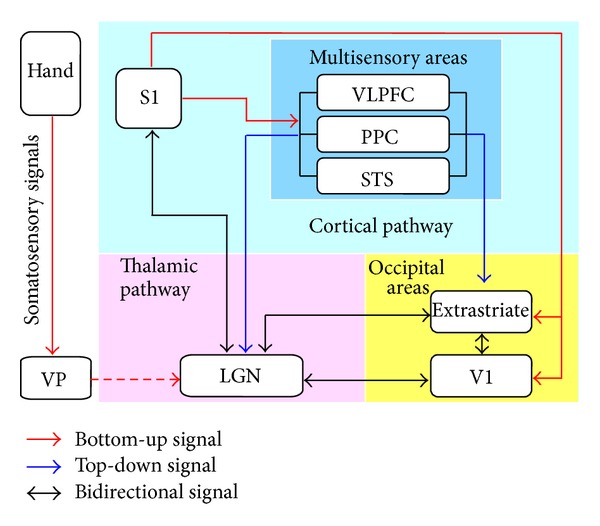
Schematic of neural pathways that convey somatosensory signals into visual areas. The solid line represents the existing connections in the normal sighted animals or human; dash line represents the rewired connections after early visual deprivation; arrows with red, blue, and black color represent the bottom-up, top-down, and bidirectional somatosensory signals, respectively. LGN: lateral geniculate nucleus; PPC: post parietal cortex; S1: primary somatosensory cortex; STS: superior temporal cortex; VLPFC: ventral lateral prefrontal cortex; V1: primary visual cortex; VP: ventral posterior nuclei.

As discussed above, much evidence supports the cross-modal processing of nonvisual signals in the visual cortex. It is then important to understand how the nonvisual sensory information reaches the occipital areas, especially in the CB subjects who have no visual experience about the external world. The candidate pathways include the thalamooccipital and corticooccipital pathway, which is categorized in Figures 1 and 2.

### 3.1. Thalamooccipital Pathway

The thalamus is an important relay that receives afferents from different sensory organs and sends efferents to the primary sensory cortex. Under normal condition, the thalamic nuclei are relay stations via which sensory information from the peripheral sensory receptors can reach the primary sensory cortex. For example, the lateral geniculate nucleus (LGN), the “visual” thalamic relay, mainly transfers visual signals to the primary visual cortex (V1); the medial geniculate nucleus (MGN) is the “auditory” thalamic relay that connects the inferior colliculus (IC) and the primary auditory cortex (A1); and the ventral posterior nuclei (VP) is the “somatosensory” thalamic relay that receives tactile signals and transmits them to the primary somatosensory cortex (S1) [120, 121]. Furthermore, the thalamus can also receive feedback signals from the sensory cortex [122–125] and the associate cortex [126–132] to modulate the input signals. It is hypothesized that nonvisual signals may bypass the traditional sensory pathway and “rewire” into the “visual” thalamus and project to the V1. This hypothesis is supported by the findings that the LGN receives rewired auditory projections from the inferior colliculus [133–139] and the MGN [140], receives rewired somatosensory projections from the VP [140, 141], and then projects efferent fibers to the visual areas in enucleated animals since birth [142]. 

Recent findings have shown that the LGN and its output projections to the V1 are atrophied in early blind subjects [24, 26, 27], which seems to contradict with this hypothesis. One putative explanation is that the atrophy of the LGN and the retinofugal pathway is the consequence of the interaction between disused neurodegeneration of the “visual” part of the pathway and cross-modal plasticity of the “rewired” nonvisual part of the pathway. In fact, pervious animal experiments have shown that the visual projections to the dorsal lateral geniculate nucleus are dramatically reduced in blind animals, while the auditory projections to the same region are strengthened [133, 142]. Another possibility is that the nonvisual signals bypass traditional retinofugal pathway (from the LGN to the V1) and pass through the pulvinar-occipital pathway [143–146], or from the LGN to the higher visual areas (such as the hMT+ and V4) [147–150]. In agreement with this interpretation, a recent fMRI study in monkeys demonstrates that direct LGN projections to the extrastriate cortex have a critical functional contribution to blindsight with V1 lesions [151]; a human study shows a direct anatomical connection between the thalamus and the hMT+ complex, that would directly convey motion information to the hMT+, thereby bypassing the V1 [147].

### 3.2. Corticooccipital Pathway

An alternative pathway is that the visual areas receive nonvisual sensory information through corticooccipital connections between these sensory modalities [140, 152, 153]. These corticooccipital connections can be further subdivided into direct corticooccipital connections and indirect polysynaptic corticooccipital connections. The former has been found between the A1 and visual cortex in adult Mongolian gerbils [154], cats [155], primates [153, 156, 157], humans [158], and congenitally blind opossums [140], and between S1 and V1 in enucleated opossums [140]. The latter has been indicated by studies showing multisensory processing in the association cortices [159], such as the posterior parietal area (PPA) [160, 161], superior temporal sulcus (STS) [162–164], ventral lateral prefrontal cortex (VLPFC) [165–167], and extrastriate areas [155, 168, 169]. The dense anatomical connections between multisensory areas and both the visual and nonvisual sensory cortices have been identified in both animal and human studies [155, 159, 163, 170, 171]. 

The corticooccipital pathway hypothesis is also supported by task-based fMRI studies [165, 172] and a resting-state fMRI study [173] in sighted subjects, an effective connectivity study [174], and TMS studies in sighted and EB subjects [23, 78, 175, 176]. In a recent study by our group, we found the rsFC between the early visual areas and S1 was dramatically decreased, while those between the higher-tier visual areas and S1, and between the early and higher-tier visual areas, were relatively preserved or even strengthened in the CB. Our findings support the hypothesis of the indirect corticooccipital pathway mediating nonvisual sensory information to the early visual areas via the relay of higher-tier ones [17]. 

It should be noted that these two pathways cannot be absolutely segregated in the brain network. They are interacted with each other by feed forward and feed back projections. For example, the auditory signals can first feed forward to the A1 for initial processing, and then feed back to the MGN and multimodal thalamic nuclei (e.g., the pulvinar) [177], and finally project to the V1 (cortico-thalamooccipital pathway). The multisensory areas such as the PPA and VLPFC also project efferents to the multimodal thalamus (the pulvinar and medial dorsal nucleus) [127–131], so they can convey the modulated nonvisual signals to the occipital area via the thalamus (Figure 1). 

The existing thalamo-cortical and corticooccipital pathways found in the normal adult animals and humans provide anatomical evidence of the supramodal nature of the visual cortex. Auditory and tactile information can be conveyed to the occipital areas through the direct and indirect connections. This anatomical connections pattern can explain the cross-modal involvement of visual cortex by nonvisual sensory tasks; furthermore, it can explain the development of a portion of the visual areas does not depend on the visual experience because inputs from other sensory modalities are sufficient to support the development of these functional patterns.

## 4. Rewiring versus Unmasking of the Nonvisual Pathway after Visual Deprivation

Two competing hypotheses have been proposed to explain the neural mechanisms of cross-modal plasticity after early visual deprivation. According to the rewiring hypothesis, cross-modal brain responses are mediated by the formation of new pathways in the sensory deprived brain. For example, after experimental destruction of the superior colliculi and the visual cortex in neonatal hamsters, the authors observed a strong projection from the retina to the A1, which can “perceive” the visual information [178, 179]. Studies in animals have also shown that when the brain is deprived of peripheral visual input at an early age, auditory inputs are re-routed to the visual cortex via the thalamooccipital pathway [133, 139–141]. However, this subcortical rewired pathway is questioned because of the lack of *in vivo* evidence. In contrast, there are considerable studies showed that the whole segments of retinofugal pathway, including the optic tract, the LGN, and optic radiation, suffered atrophy and loss of integrity in humans after early visual deprivation [16, 23, 27].

The unmasking hypothesis proposed that the loss of a sensory input induces unmasking and/or strengthening of the existing neural pathways. As discussed in Sections 3.1 and 3.2, the tactile and auditory inputs can be conveyed to the visual cortex via the existing thalamooccipital pathway or corticooccipital pathway that have been confirmed in normal adult animals and humans. Generally, these nonvisual signals can modulate the processing of visual information in sighted subjects [180]; however, they cannot induce subjective nonvisual sensations and occipital activation due to being masked by the dominant visual input [40, 64, 176]. The occasional findings of occipital processing nonvisual signals might be task-dependent that dramatically reduced the masking effects of the visual input [40, 52, 55]. However, after early visual deprivation, nonvisual processing in the visual cortex is strengthened or unmasked because of the lack of visual input. The unmasking hypothesis is also supported by the cross-modal responses after short-term visual deprivation (blindfolding). Several hours to days blindfolding resulted in rapid, reversible improvement in task performance and recruitment of the visual cortex in nonvisual processing, such as tactile discrimination [181, 182], Braille reading [183], and sound localization [184, 185]. Rapid cross-modal responses exclude the possibility that these are mediated by the establishment of new anatomical connections. This claim was also supported by sensory substitution devices (SSD) studies that the visual cortex was also involved in processing nonvisual tasks after a short period of SSD training in sighted subject [50, 72, 186]. It is possible that a short period of blindfolding and SSD training unmasks and strengthens pre-existing connections between the nonvisual and the occipital cortices.

## 5. Stimulus-Driven or Top-Down Control of the Nonvisual Processing in the Visual Cortex

As shown in Figure 1, nonvisual signals can reach the visual cortex via thalamooccipital pathway, corticooccipital pathway, or combinations of these two pathways. An important but unsolved question is the nature of these nonvisual signals: stimulus-driven or top-down. The stimulus-driven signals refer to those from sensory organs or early sensory cortex, whereas the top-down signals are refer to those who came from the higher-level cortical regions. Clarifying this question can help us to understand the neural mechanisms underlying cross-modal recruitment of the visual cortex and to design appropriate interventions to improve the adaptive capacity of blind subjects to the external environments. 

### 5.1. Stimulus-Driven Hypothesis

The following evidence supports stimulus-driven nature of the nonvisual signals that reach the visual cortex. These nonvisual signals can be conveyed from sensory organs to the visual cortex via rewired thalamic-cortical pathway [133–139] and those from the nonvisual primary sensory cortices via the direct corticooccipital connections (such as from A1 to V1) [153, 156, 157], which bypass the higher-tier “cognitive” cortex, so the inputted nonvisual signals may not be modulated and reflect the pure stimulus-driven information. The involvement of the visual cortex in nonvisual perception in both sighted [45, 46, 72] and EB [37, 42, 50, 61–63, 66, 68, 69, 80] subjects also suggests the stimulus-driven nature of these nonvisual signals because the top-down effects such as visual imagery and attention were well controlled in these studies. The event-related potentials (ERPs) studies demonstrated that the N1 component of occipital response following nonvisual stimulation was as early as the typical component of the visual perception in EB subjects [76, 77, 187]. For example, a recent report showed that the shape-selective activity in the LOC was present as early as 150 ms following the onset of tactile stimulation, which support the stimulus-driven somatosensory input to the LOC [187]. 

### 5.2. Top-Down Hypothesis

According to the top-down mechanism, the peripheral auditory/tactile signals are firstly modulated and refined by the higher-level cortical regions, such as the multisensory associate areas (VLPFC, PPA, and STS), and then feed back to the visual cortex through the indirect corticooccipital pathway and cortico-thalamooccipital pathway (Figure 1). The existing anatomical feedback projections from higher-level cortical regions to the thalamus and visual cortex support this top-down mechanism [132, 155, 159, 163, 170, 171]. Furthermore, many task-evoked and lesion studies have demonstrated the modulation effects of the fronto-parietal network on the thalamus and visual cortex [165, 188–191]. It seems that in CB subjects, the top-down attention modulation of the occipital activity was strengthened [75, 96, 192–195]. Visual imagery, a complex mental process, can also activate the specific visual areas that are usually recruited by certain visual properties (shape, space, and color), which supports the top-down controlling the visual activities [102–108]. It should be noted that the earlier ERP response cannot exclude the effects of top-down modulation, because top-down attention can also module the early components of ERP, for example, the N1 negativity or even earlier peak [196, 197]. Furthermore, a ERP study demonstrated that the top-down attention modulation of the occipital activity was significantly strengthened in EB subjects [75]. The following paragraphs state the two popular cognitive processes (visual imagery and attention) that might contribute to the top-down recruitment of the visual cortex.

#### 5.2.1. Visual Imagery

Visual imagery mechanism supports the hypothesis of top-down recruitment of the visual cortex because the mental progress can recruit the visual cortex [102–108]. For example, Kosslyn et al. showed that the visual areas 17 and 18/19 were activated by a stripe imagery task, and rTMS delivered to area 17 did disrupt both visual perception and imagery performance [104]. This study indicates that the early visual areas are involved in at least some forms of visual imagery as well as in visual perception [104]. It is interesting to noted that not only the early visual area, but also the higher-tier ones can specifically respond to the visual imagery task, including the ventral stream for shape imagery [47, 198–200], and the dorsal stream for motion and spatial imagery [107, 108, 201–203], which highly corresponded with the hierarchy representation of the visual perception. De Volder showed that in both sighted and EB subjects, auditory triggered mental imagery of shape can also activate the ventral occipitotemporal and visual association areas [200].

#### 5.2.2. Attention Controlling

Top-down attention may be the most extensively studied cognitive process that can modulate the activation of the visual cortex [204–208]. It is proposed that visual attention modulates visual processing even at an early stage; it not only modulates the gain on incoming visual information, but also adds a pure top-down signal that increases baseline activity in the visual cortex; moreover, attentional modulation can exert on different aspects of visual perception, such as locations, features, objects, or a combination [208]. Furthermore, not only the visual attention, but also the nonvisual attention can also activate the visual cortex [207, 209–211]. 

In the CB subjects, electrophysiological or neuroimaging studies have revealed that the top-down attention modulation was strengthened when they performing tactile/auditory attention-demanding tasks [75, 111, 112, 212–215]. Additionally, the occipital cortical areas were activated in the CB subjects by attention tasks through nonvisual modalities [60, 96, 194, 216, 217]. In combination with evidence of attention modulation on visual perception in the SC [218–220], these findings indicate increased top-down attention modulation of occipital activity in the CB. 

In summary, the nature of the nonvisual signals to the visual cortex may be either stimulus-driven or top-down, or both. Indeed, effective connectivity analysis offers evidence for the coexistence of both bottom-up and top-down information flows during nonvisual perception [221–223]. 

## 6. Conclusions

The cross-modal processing of nonvisual signals in the occipital areas in both sighted and EB subjects suggests that the functional organization of the visual cortex is supramodal in nature. The cross-modal plasticity can also account for parts of the findings in the EB subjects. The normally existing thalamo-cortical and corticooccipital pathways provide anatomical evidence for the supramodal nature of the visual cortex. The cross-modal plasticity in the EB might be driven by two neural mechanisms: rewiring and unmasking, although there is lack of *in vivo* evidence for the former. Further studies with more advanced *in vivo* imaging techniques should be implemented to clarify this issue. Finally, the nature of the nonvisual signals to the visual cortex may be either stimulus-driven or top-down, or both. Further understanding the issue may help us to design appropriate interventions to improve the adaptive capacity of blind subjects to the external environments.
